# Memory-assisted reinforcement learning for diverse molecular de novo design

**DOI:** 10.1186/s13321-020-00473-0

**Published:** 2020-11-10

**Authors:** Thomas Blaschke, Ola Engkvist, Jürgen Bajorath, Hongming Chen

**Affiliations:** 1 Hit Discovery, Discovery Sciences, R&D, AstraZeneca Gothenburg, Mölndal, Sweden; 2 Centre of Chemistry and Chemical Biology, Guangzhou Regenerative Medicine and Health-Guangdong Laboratory, Science Park, Guangzhou, China; 3 Department of Life Science Informatics, LIMES Program Unit Chemical Biology and Medicinal Chemistry B-IT, Rheinische Friedrich-Wilhelms-Universität, Endenicher Allee 19c, Bonn, 53115 Germany

**Keywords:** Deep learning applications, Reinforcement learning, De Novo design, Exploration strategy, Recurrent neural networks

## Abstract

In de novo molecular design, recurrent neural networks (RNN) have been shown to be effective methods for sampling and generating novel chemical structures. Using a technique called reinforcement learning (RL), an RNN can be tuned to target a particular section of chemical space with optimized desirable properties using a scoring function. However, ligands generated by current RL methods so far tend to have relatively low diversity, and sometimes even result in duplicate structures when optimizing towards desired properties. Here, we propose a new method to address the low diversity issue in RL for molecular design. Memory-assisted RL is an extension of the known RL, with the introduction of a so-called memory unit. As proof of concept, we applied our method to generate structures with a desired AlogP value. In a second case study, we applied our method to design ligands for the dopamine type 2 receptor and the 5-hydroxytryptamine type 1A receptor. For both receptors, a machine learning model was developed to predict whether generated molecules were active or not for the receptor. In both case studies, it was found that memory-assisted RL led to the generation of more compounds predicted to be active having higher chemical diversity, thus achieving better coverage of chemical space of known ligands compared to established RL methods.

## Introduction

Over the last few years, machine learning, and in particular deep learning, has led to numerous breakthroughs in the field of computer vision, speech recognition, and medical diagnosis. In the regime of big data, some machine learning models have surpassed the human level of accuracy. One of the most popular example is the development of AlphaGo, a deep learning architecture capable of winning against one of the best human players in the game Go [1]. Also, more and more machine learning systems yield fast and accurate diagnosis of life-threatening conditions such as strokes and detection atrial fibrillations, and some of their algorithms have been approved by the FDA [2]. In the field of drug discovery, machine learning is often applied for property prediction of chemical structures. In some instances, deep learning has further increased the performance of prediction methods such as support vector machines and random forests in multi-task scenarios [3], in addition some studies indicate that deep learning models are superior in handling missing data while training [4]. Most of the recent studies use established molecular representations such as extended-connectivity fingerprints (ECFP) [5]. However, specialized deep learning architectures like Mol2Vec [6], DruGAN [7], GraphConv [8] allow the extraction of molecular representations based on the input of a molecular graph or using the SMILES notation; in a few reported cases reported this approach leads to some incremental improvements for property prediction over standard representations such as ECFP [9].

Accurate properties prediction is often a crucial step in a drug discovery project, especially when the concurrent optimization of multiple properties, such as physiochemical properties, pharmacokinetic profile, activity against a biological target, or selectivity is attempted. The search for compounds with a specific set of properties is a non-trivial task and a slow and costly process even when it is performed in silico because chemical space is vast. In order to avoid extensively searching chemical space, one would ideally aim to generate compounds with desired properties and avoid enumerating exceedingly large numbers of compounds [10].

In addition to more accurate property prediction, some deep learning architectures allow the generation of novel molecules and are thus used for de novo design [11]. The potential of molecule generation has been shown in different studies, and multiple architectures and strategies have been devised for the generation of compounds. A number of architectures, such as variational autoencoders [12], recurrent neural networks (RNNs) [13], conditional RNNs [14], and generative adversarial networks (GANs) [15] have been proven successful in generating molecules. A popular approach for generative modeling is so-called reinforcement learning (RL) using RNNs. RL allows to couple generative models with an arbitrary property predictor to direct the generative model towards a specific subset of the chemical space where most of the compounds meet pre-specified properties. For example, Olivecrona et al. proposed the REINVENT algorithm which combined RNNs and RL to generate molecules that are enriched for chemical and biological properties [16]. In their retrospective study, the authors showed that it is possible to rediscover known experimentally validated ligands using RL, which neither the generative model nor the prediction model had been trained on. Benjamin et al. exploited the GAN for a sequence generation model to generate molecules with multi-objective reinforcement learning (named ORGANIC) [17]. Putin et al. extended the ORGANIC framework using differential neural computer (DNC) [18, 19]. The results showed that the DNC-based GANs generated SMILES that were longer, more diverse, and more complex than the SMILES generated by ORGANIC. Gupta et al. used a RNN in combination with transfer learning to perform target specific fine tuning of the generative model [20]. Different from many purely computational studies, only few studies reporting generative modelling have also included synthesizes and experimental evaluation of novel compounds. For example, Merk et al. applied transfer learning to six different nuclear receptors followed by in vitro validation of the de novo designs, in which four out of five ligands displayed bioactivity [21]. Zhavoronkov et al. used a two-step RL approach (GENTRL) to design novel ligands for discoidin domain receptor 1 and test their bioactivity [22]. Four compounds were active in biochemical assays, and two were validated in cell-based assays. These results suggest that generative modelling can be applied in prospectively in de novo design.

Even though it is possible to generate novel compounds with the desired properties, the resulting solutions often lack chemical diversity [23–25]. Deursen et al. proposed to address this issue with the introduction of Generative Examination Networks (GEN), which perform statistical analysis of the generated compounds during training [26]. However, their study does did not include the application of this approach in any pre-defined optimization scenario. Typically, in a given optimization scenario, the model finds a particular solution which only consists of molecular scaffolds. This is caused by the so-called mode collapse or policy collapse from which the RL and the GAN models suffer [27–30]. In this case, once the model finds a good solution with desired properties, it keeps sampling this particular section of space without exploring alternative sections. This problem is mostly unsolved so far, and, to the best of our knowledge, only Liu et al. [31] have attempted to engineer a complex RNN model that includes a normal RNN model for structure generation and an explorative RNN model for enforcing the exploration of alternative solutions. However, even with fine-tuning, the method did not significantly increase the diversity of generated structures compared to the REINVENT method.

Herein we introduce a novel sampling strategy for RL with RNNs, which utilizes the so-called memory unit to enable modifying the output of any predictor such that RL can move away from an already explored section of chemical space. The memory unit uses well-established similarity measures such as Tanimoto similarity of compounds or scaffolds to compare samples of chemical space and enables a flexible yet intuitive way to fine-tune available RL architectures.

## Methodological overview

The memory unit is a separate module that can be used in combination with any property predictor for RL. In this study, we chose to use the memory unit in combination with the REINVENT [16, 32] approach. The REINVENT methodology includes two coupled generative neural networks, namely a “prior” network and an “agent” network for structure generation in a stepwise manner. During the first stage, the prior network is trained to generate novel compounds that are similar to ChEMBL [33] compounds. After the network is trained, the agent network is initialized with the same parameters as the trained Prior network. In the second stage, the agent network is redirected through RL to optimize structures using the property predictor as the scoring function in combination with the likelihood of molecular structure in the Prior model and the reward score from a memory unit.

### Memory-assisted reinforcement learning formulation

In REINVENT algorithm, compounds are represented by SMILES strings. The scoring of a compound is dependent on two functions, S(c) and M(c), where S(*c*) is the scoring function that evaluates the desirability of a generated compound *c* using some arbitrary method, and the M(*c*) is the output score from the newly introduced memory unit. The goal of the RL is updating the agent network from the prior network to increase the expected score for the generated compound. However, we would like our agent to be rooted to the prior, which has learned the syntax of SMILES and the distribution of molecular structures in ChEMBL. We, therefore, denote an augmented likelihood “logP(*c*)_Aug_” as a prior likelihood modulated by the desirability of a sequence:

1 $$logP\left( c \right)_{Aug} = logP\left( c \right)_{PriorNetwork} + \sigma \times S\left( c \right) \times M\left( c \right)$$

where σ is a scalar coefficient, S(*c*) the output of the scoring function, and M(*c*) the output of the memory unit, which is either 0 or 1. The total reward R(*c*) of a molecule *c* can be seen as the agreement between the likelihood of the Agent model “logP(*c*)_AgentNetwork_“and the augmented likelihood:

2 $$R\left( c \right) = \left( {logP\left( c \right)_{Aug} - logP\left( c \right)_{AgentNetwork} } \right)^{2}$$

The goal of the Agent is maximizing the expected reward, which is achieved by minimizing the loss function:

3 $$loss = - R\left( c \right)$$

### Memory unit

**Fig. 1:**
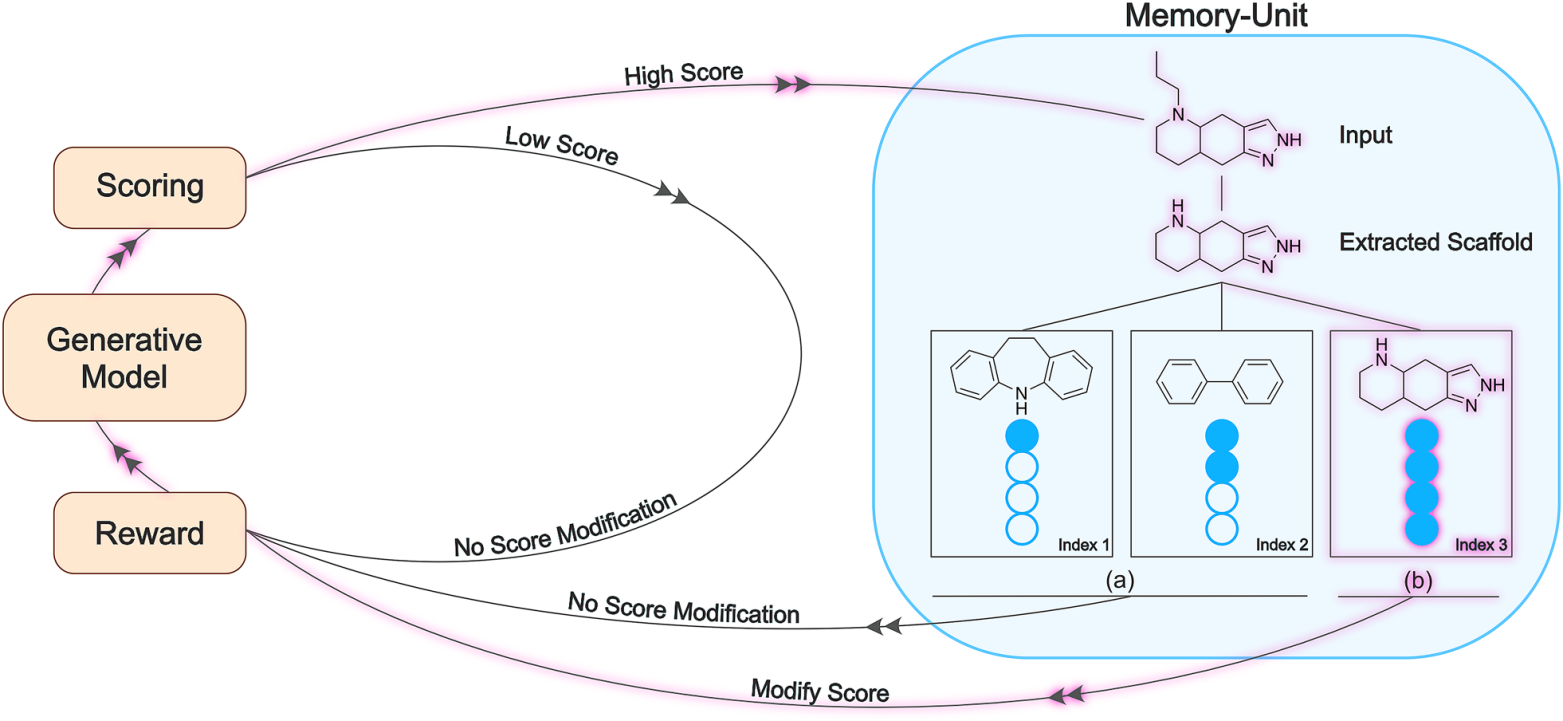
Schematic workflow of the memory unit. The memory unit (left) is integrated into the regular RL cycle (right). The generative model produces structures, which are scored by an arbitrary scoring function. Only molecules with a high score are processed by the memory unit. Every input molecule is compared to all indexed compounds based on their molecular scaffold or their fingerprint. If the generated scaffold matches an indexed scaffold or the fingerprint similarity is greater than a defined value, the input molecule gets added to the corresponding index-bucket pair. If the buckets are not filled, shown in (**a**), the memory unit does not alter the scoring. If the bucket is full, illustrated in (**b**), the score is modified, and the generative model has to explore new chemical structures. For an exemplary compound, the path of structure generation is highlighted. Because the bucket for the corresponding scaffold is filled, the score of this compound is modified

**Fig. 2:**
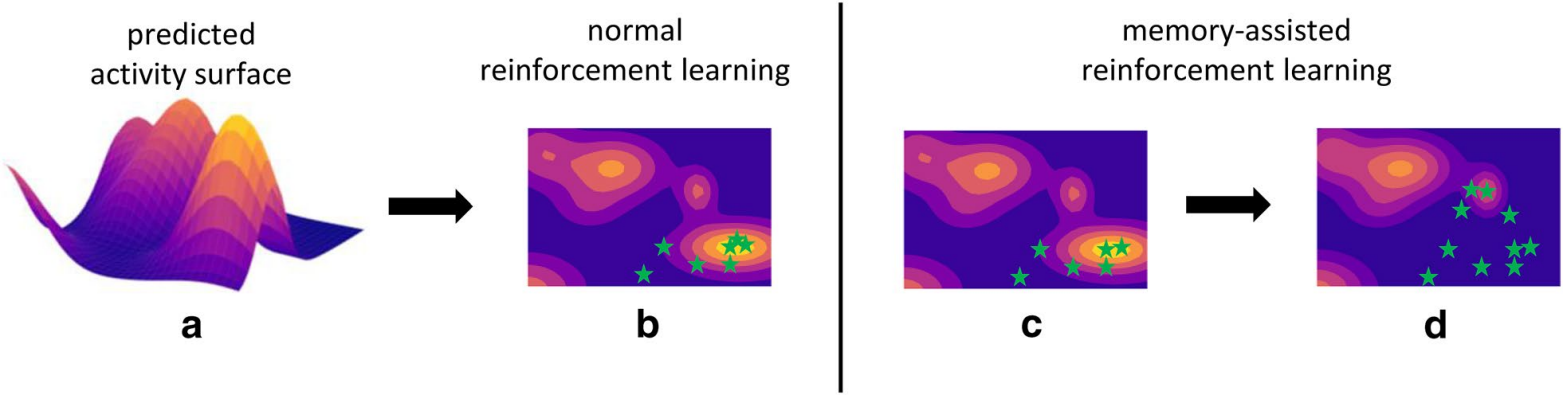
Schematic comparison of regular and memory-assisted reinforcement learning utilizing a QSAR model. **a** The activity prediction surface of a non-linear QSAR model is illustrated. A generative model iteratively constructs compounds (green stars), which are predicted to be active. **b** Using regular reinforcement learning, the model generates only compounds of the first local maximum it reaches. **c** Memory-assisted reinforcement learning starts with regular reinforcement learning. **d** Once the chemical space is locally explored, the memory alters the prediction surface and forces the generative model to find a new local maximum

The memory unit is designed to be incorporated into the RL scoring function such that the generative model is not only exploring a given favorable area in chemical space, leading to a high score, but many different regions of chemical space. To do so, the memory unit keeps track of all highly scored molecules during RL. If a molecule in the current batch is very similar to the saved compounds in the memory unit, its M(*c*) score of the memory unit is set to zero. In this way, the memory unit effectively alters the surface of the scoring function so that the gained reward for the generated molecules is minimal, and the neural network is discouraged to continue generating similar molecules. The integration of the memory unit into a generative model is illustrated in Fig. 1. Furthermore, Fig. 2 illustrates the influence of the memory unit on RL. In traditional RL, the model can generate highly scored compounds. Once it is at a local maximum for the reward, the model is often unable to find a different solution as it would need to exit the local maximum again. Accordingly, the reward would need to be decreased in order to find other local maxima. This situation ultimately leads to the so-called policy collapse. RL will generate very similar solutions that are not substantially different from each other. In other words, RL generates different compounds with the same scaffold where only the position of a single substitute is changed. The memory unit is introduced to address this issue. Using the memory unit, RL will reach the same local maximum for the reward. However, RL will be prohibited to exploit the given region of chemical space indefinitely. After a specified number of generated compounds have been saved in the memory unit, the memory unit resets the reward and penalizes generated compounds that are similar to the compounds saved in the memory unit. By doing so, RL is enabled to exit a specific local maximum and reach the next local maximum in a different region.

The implementation of the memory unit is based on a hash table, which consists of multiple index-bucket pairs. A bucket contains a fixed number of chemical structures that belong to the same cluster, and the index corresponds to the seed chemical structure of the bucket. The molecules with high S(*c*) scores in a batch are taken as query structures to compare with all index structures in the memory unit. The memory score M(*c*) is related to the query structure from the batch and the number of molecules that are stored in the memory. The default bucket size is 25, which means that the bucket can store a maximum of 25 compounds. If a query compound is similar (Tanimoto similarity index higher than a pre-defined cutoff value) to the index compound and the bucket is full, the memory score is set to 0 for the query molecule and it is rejected. If the bucket is not fully occupied, the memory score is set to 1 and the query compound is added to the bucket. If the highly scored compound is not similar to any index structure in the memory unit, a new index–bucket pair is created with the new compound. Batch compounds with low S(*c*) scores are not compared to the index molecules of the memory unit and their M(*c*) score set to 1.

The assessment of chemical similarity has, therefore, a profound effect on how the memory unit alters the reward. We evaluated four different criteria for chemical similarity. First, we considered the Jaccard index [34] for binary vectors, also known as Tanimoto similarity, on the basis of the ECFP4 fingerprint as a molecular representation. Each time a highly scored compound was generated, Tanimoto similarity to all index compounds was calculated. By default the similarity cutoff value was set to 0.6, if the similarity values were equal to or larger l than 0.6 and the bucket was not full, the molecule was added. If no similarity value to an index compound was at least 0.6 or larger a new index-bucket pair was created.

Furthermore, similarity can not only be evaluated at the compound level but also at the scaffold level. In this case, similarity was assessed either on the basis of Bemis-Murcko (BM) scaffolds [35] or the corresponding carbon skeletons. A carbon skeleton is obtained from a BM scaffold by replacing all heteroatoms with carbos and setting all bond orders to 1.

In the BM scaffold case, for each highly scoring compound having a score of at least 0.6, the BM scaffold was extracted and compared to all BM scaffolds in the index of a hash table. If an exact match for the BM scaffold was found, the compound was added to the respective bucket. If the BM scaffold was previously unobserved, a new index-bucket pair was created with the BM scaffold as an index, and the corresponding compound was added to the bucket. If a bucket already contained 25 compounds, the memory index returned a value of 0; otherwise, a value of 1 was returned. The same protocol was also applied to matching identical carbon skeletons.

In addition to exact carbon skeleton matching, we also evaluated a fuzzy skeleton filter. The fuzzy scaffold filter was based on the carbon skeleton but used the atom pair fingerprint [36] for similarity assessment to compare carbon skeletons instead of exact scaffold match. For each generated carbon skeleton, an atom pair fingerprint was calculated, and different carbon skeletons were compared using the Tanimoto coefficient. If the coefficient value was at least 0.6, the two scaffolds were considered similar and the corresponding compound was added to the same bucket.

### Retrospective validation

To test the effectiveness of the memory unit with different similarity assessments, we used two different strategies to generate optimized sets of compounds.

### Optimize structures towards a specific LogP

As a proof of concept, we tried to optimize the generated compound toward a desired LogP range. LogP is an important parameter in pharmaceutical research as it indicates the balance between solubility and cell permeability of a molecule. Usually, in early-stage drug development, LogP values ranging from of 2–3 are often considered to be desirable for candidate compounds. Therefore, we set our scoring function to return the value of 1.0 if the calculated LogP is between 2 and 3. If the values are not in that range, we calculate a score as following:

4 $$S = 1 - {\text{tanh}}\left( {\hbox{min} \left( {\left| {2 - AlogP} \right|, \left| {3 - AlogP} \right|} \right)} \right)$$

where *S* is the scoring function with values between 0.0 and 1.0, “tanh” the hyperbolic tangent, and AlogP is the calculated LogP value using Crippen’s atom-based LogP calculation approach [37] implemented in RDKit [38].

### Optimize structures for high predicted activity against given biological targets

As another validation, we generated molecules that were predicted to be active against 5-hydroxytryptamine receptor type 1A (HTR1A) and the dopamine type 2 receptor (DRD2). First, for each target, we extracted bioactivity data and assembled a training, validation, and test set. Next, we trained support vector machine (SVM) models [39] using the respective training sets and optimized the parameters on the validation sets. The test sets were held back to obtain an estimation for the prediction performance of the SVM models. In the next step, we used the probabilistic output of the trained SVM classifiers as the respective reward function for molecule generation.

### Data sets

The bioactivity data for HTR1A and DRD2 were extracted from ExCAPE-DB [40]. The database contains a total of 3599 actives (pIC50 > = 7) and 66,684 inactive (pIC50 < 7) compounds for HTR1A and 2981 actives (pIC50 > = 7) and 346,206 inactive (pIC50 < 7) compounds for DRD2 respectively. For both data sets, all actives were selected. For HTR1A all inactive compounds were selected and for DRD2 a subset of 100,000 inactive compounds was randomly selected. To decrease the nearest neighbor similarity between the training and testing structures [41] the actives were grouped using the Butina clustering algorithm [42] and the Tanimoto similarity calculated based upon of the extended connectivity fingerprint with bond diameter of 6 (ECFP6) [5]. According to the Butina algorithm, clusters were created by selecting molecules as centroids and assigning every compound with a similarity higher than a defined similarity cutoff to this cluster. In our analysis, we chose a Tanimoto similarity cutoff of 0.4. The centroids were selected, such that the number of molecules that were assigned to any cluster was maximized. After the compounds were assigned to their respective clusters, the clusters were sorted by size and iteratively assigned to the test, validation, and training set, such that the final distribution of actives in the test, validation, and training set was 20%, 20%, and 60%, respectively. The inactives were randomly assigned to the three sets using the same ratios.

### SVM bioactivity models

The two non-linear SVM classifiers were built using scikit-learn [43] on the training sets as predictive models for DRD2 or HTR1A activity, respectively. The compounds were encoded as count-based ECFP6 and folded into 2048 dimensional vectors using a modulo operation. The optimal c value and class weights in the final models were obtained from a grid search for the highest MCC [44] performance on the respective validation set. After the determination of the optimal hyperparameter, new SVM models were trained and calibrated using Platt scaling [45] to obtain probabilistic classification values between 0 and 1. The MinMax kernel was used [46]. For c, grid search values between 10^−5^ and 10^−5^ were evaluated by incrementally changing the exponent by 1. Uniform class weights and class weights inversely proportional to class frequencies were considered. The test sets were only used to estimate the “real” performance on unknown compounds that were not used for the hyperparameter search nor the initial training of the SVMs.

### Generative model

As a generative model, we used a RNN similar to the one reported in REINVENT [16]. The generative model featured an embedding layer followed by three gated recurrent units (GRU) [47] with 256 dimensions, and finally a linear layer that reshaped the outputs to the size of all possible tokens. The loss function is the Negative Log-Likelihood (NLL):

5 $${\text{loss}}\left( {{\text{sequence}}} \right) = \sum\nolimits_{t = 1}^T {\log {\text{P}}\left( {{{\text{x}}_{\text{t}}}{{\text{x}}_{{\text{t}} - 1}}, \ldots ,{{\text{x}}_1}} \right)}$$

where “x_t_” is a random variable representing the probability for all possible tokens of the vocabulary at step “t” and “x_t-1_” is the token chosen at the previous step.

To train the generative model, the so-called prior model, we used a more general dataset that did not contain known active molecules for HTR1A and DRD2. We extracted all compounds from ChEMBL 25 and removed all compounds with more than 50 heavy atoms. Furthermore, we removed all stereochemistry information and canonicalized the SMILES strings using RDKit. Additionally, we filtered the ChEMBL compounds for the known HTR1A actives and based on the similarity to the DRD2 actives extracted from ExCAPE. All 3599 HTR1A actives and compounds with an ECFP4 Tanimoto similarity of 0.4 or more to any of the 2981 DRD2 actives were excluded. This resulted in a final dataset of 1513,367 unique compounds, which were used to train the prior model for ten epochs using the Adam optimizer [48] and a learning rate of 0.01.

## Results

### LogP optimization

As the LogP of a compound is an important indicator for membrane permeability and aqueous solubility of potential drug candidates, a common task in a drug discovery project is to optimize the LogP of a compound series while maintaining the overall characteristics of the series. In our first proof-of-concept study, we replicated this task by optimizing the LogP of known DRD2 inhibitors with high LogP values.

To restrict the prior model to a set of known bioactive molecules, we selected 487 known DRD2 compounds from ExCAPE with a LogP of larger than or equal to 5 and applied transfer learning to the prior model. The model was retrained on these 487 compounds for 20 epochs directing it to produce DRD2 compounds with a high LogP. The next step was the RL to force the bias before generating molecules with a LogP between 2 and 3. During RL, the model created 100 compounds per iteration that were scored based on their LogP value. RL was applied for 150 iterations, such that a total of 15,000 compounds were generated. We investigated four different similarity measures: one at the compound level and three different similarity measures at the scaffold level. Table 1 summarizes the number of generated optimized compounds with a LogP of 2.0–3.0. All different types showed an increase in the number of generated compounds and generated BM scaffolds and carbon skeletons.

**Table 1:** Models for optimized LogP using reinforcement learning

Target	Memory type	Generated optimized compounds	Unique BM scaffolds	Unique carbon skeletons
LogP	No memory	938	727	396
	Compound similarity	3451	2963	1472
	IdenticalBMScaffold	3428	2865	1398
	IdenticalCarbonSkeleton	3315	3002	1799
	ScaffoldSimilarity	3591	3056	1538

The generative models were tuned for generating compounds with a predicted LogP between 2.0 and 3.0 using RL for 100 iterations. During each iteration, a model generated 150 compounds resulting in a total of 15.000 compounds. Only compounds with a predicted LogP between 2.0 and 3.0 were retained

In the case of the RL without the memory unit, the model was able to generate 938 unique compounds with a predicted LogP between 2 and 3. This resulted in 727 different BM scaffolds and 396 carbon skeletons. The use of the memory unit increased the number of generated optimized compounds by threefold. With 3591 generated compounds, the memory unit matching BM scaffold sampled most compounds. Not only the number of generated compounds, but also the number of generated BM scaffolds and carbon skeletons increased using the memory unit.

However, as stated at the beginning, a LogP optimization resulting in compounds with unknown scaffolds would be undesirable as one would like to maintain the bioactivity of compounds containing the known scaffolds. To analyze if the use of the memory unit resulted in the generation of unrelated compounds to the training set, we investigated analog relationships between the generated compounds with the training set using count-based ECFP6 Tanimoto similarity and the matched molecular pair (MMP) formalism [49]. We fragmented the generated and the training set molecules applying a size restriction such that the larger compound fragment (also referred to as MMP–core) was at least twice as large as the other fragment [50]. The obtained MMP-cores were then compared to the MMP-cores of the training set compounds. The results are shown in Table 2.

**Table 2:** Generated analogs compounds with optimized LogP

Target	Memory type	ECFP6 analogs	MMP analogs	Shared MMP cores with the training set
LogP	No memory	145	6	5
	Compound similarity	421	24	16
	IdenticalBMScaffold	485	30	17
	IdenticalCarbonSkeleton	474	27	19
	ScaffoldSimilarity	549	38	18

The generated structures with predicted LogP between 2.0 and 3.0 were compared to the 487 training compounds based on fingerprint similarity (counted ECFP6) and their MMP relationships. Generated compounds with a Tanimoto similarity of 0.4 or higher were considered analogs. The generated compounds were fragmented, and MMP relationships with training compounds were explored. If an MMP-core was present in the set of generated structures and the set of training compounds, it was considered a shared MMP core

Using RL without the memory unit, only 145 optimized compounds with Tanimoto similarity of at least 0.4 to the nearest neighbor from the training set were obtained. In comparison, up to 549 compounds were ECFP6 analogs meeting the same similarity cutoff. An equivalent trend in analog generation was observed when applying the MMP formalism. Using RL without the memory unit, the optimized compounds contained only five MMP cores from the training set. In comparison, the optimized compounds generated by the RL using the memory unit shared up to 19 MMP-cores with the training set, indicating that the memory-assisted RL led to more generated compounds, which also covered a more relevant section of chemical space compared to the RL without a memory unit.

### Optimization of compounds for high predicted activity against HTR1A and DRD2

As a second proof-of-concept study, we attempted to apply the memory-assisted RL in more complex optimization scenarios. This time we tried to generate compounds with improved predicted bioactivity. We chose HTR1A and DRD2 as targets and extracted bioactivity data from the ExCAPE database. Both targets are well-studied neurotransmitter receptors for which sufficient bioactivity data was available a to compare generated compounds at a large scale with experimentally validated compounds.

We trained and optimized non-linear SVM models using Platt scaling to obtain probabilistic activity predictions between 0 and 1. The predictive performances of the activity models are shown in Table 3.

**Table 3:** Predictive performance of the SVM models

Target	Set	Bal. ACC	ROC AUC	F1	MCC
HTR1A	Training	0.98	0.99	0.77	0.78
	Validation	0.96	0.99	0.75	0.75
	Test	0.96	0.99	0.75	0.75
DRD2	Training	0.99	0.99	0.77	0.79
	Validation	0.93	0.98	0.70	0.71
	Test	0.95	0.99	0.71	0.72

The SVM model was trained on the training set and the hyperparameters c, the choice of the kernel, and the class weight were optimized towards a high F1 score on the validation set. The test set was used to estimate the predictive performance of unknown compounds. “bal” stands for balanced

The HTR1A activity model showed excellent balanced accuracy (BA) of 0.96 for the validation and the test set. Also, the F1 [51] and Matthews’s correlation coefficient (MCC) score yielded high values of 0.75 for the validation and test set, indicating low misclassification of the active compounds. The DRD2 activity model showed a similar performance. BA reached high values of 0.93 and 0.95 for the validation and test set, respectively. For the test set, the F1 and MCC score values were 0.71 and 0.72, respectively. The area under the receiver operating characteristic curve (ROC AUC) [52] values, an important metric for ranking compounds in virtual screening and RL, were nearly optimal with 0.99 for test set of both HTR1A and DRD2.

For RL, we sampled our generative model for 300 iterations. At each iteration, the model created 100 SMILES, which were scored by the activity model and then passed to the memory unit, generating in total 30,000 compounds. For HTR1A, RL was performed with a learning rate of 0.001 whereas for DRD2 the RL was performed with a learning rate of 0.005 to accommodate for the fact that all of the known DRD2 analogs were removed from the generative model. We considered only compounds with a predicted activity > = 0.7 as active. We validated the same memory units as in the LogP optimization part (Table 4).

**Table 4:** Compounds generated using reinforcement learning

Target	Memory type	Generated active compounds	Unique BM scaffolds	Unique carbon skeletons
HTR1A	No memory	9323	7312	5446
	Compound similarity	16,779	13,304	9887
	IdenticalBMScaffold	17,390	13,863	9941
	IdenticalCarbonSkeleton	17,597	15,531	12,408
	ScaffoldSimilarity	17,383	15,296	12,082
DRD2	No memory	5143	2635	1949
	Compound similarity	21,486	17,844	12,749
	IdenticalBMScaffold	22,312	14,850	8220
	IdenticalCarbonSkeleton	22,115	19,096	12,562
	ScaffoldSimilarity	22,784	20,712	16,434

The generative models were directed towards predicting active compounds using RL for 300 iterations. During each iteration, a model generated 100 compounds resulting in a total of 30.000 compounds. Only compounds with a prediction score of at least 0.7 were considered active

Under the same experimental conditions, the number of generated compounds increased nearly twofold and more than fourfold across all different memory-types for HTR1A and DRD2, respectively. For RL with the HTR1A predictor, 9323 unique compounds were generated. Using any memory unit increased the number of generated compounds by nearly twofold, where the largest number of compounds (17,597) was generated using the identical carbon skeleton memory unit. The number of generated BM scaffolds increased by the same ratio as the compound generation for all memory units; 7312 BM scaffolds for RL without memory and 15,531 BM scaffolds using the scaffold similarity memory. Also, the number of generated carbon skeleton increased ~ twofold using the memory units. In the case of the standard RL, 5446 carbon skeletons were obtained, while 12,408 carbon skeletons were obtained using the identical carbon skeleton memory unit.

For RL with the DRD2 predictor and without a memory unit, 5143 unique compounds were generated accounting for 2635 BM-scaffolds and only 1949 carbon skeletons. In contrast, all memory-assisted RL yielded a larger number of generated compounds of more than fourfold. The largest number of compounds (22,784) was generated using the scaffold similarity memory unit. The memory-assisted RL did not only increase the number of generated compounds, but mostly also increased the number of generated BM scaffold and carbon skeletons. The number of generated BM scaffolds increased by at least fivefold in the case of the identical BM scaffold memory unit and more than a sevenfold increase in the case of the identical carbon skeleton memory unit. The number of carbon skeletons increased from fourfold up to eightfold for the identical BM scaffold memory and the scaffold similarity memory, respectively.

To investigate if the generated compounds covered a relevant region of chemical space for HTR1A and DRD2, we established and counted analog relationships for these compounds. If the Tanimoto similarity using count-based ECFP6 between a generated compound and the nearest neighbor of known compounds was at least 0.4 the compound was considered to be an analog. Additionally, for a much stricter analog definition, analog relationships between the generated compound and known were established using the size-restricted MMP formalism (Table 5).

**Table 5:** ECFP6 analogs and MMP analogs generated for the DRD2 and HTR1A data set

Target	Memory type	ECFP6 analogs		MMP analogs	
		Set		Set	
		Training	Validation and test	Training	Validation and test
HTR1A	No memory	1726	1584	70	69
	Compound similarity	2734	2742	110	97
	IdenticalBMScaffold	3703	3660	57	89
	IdenticalCarbonSkeleton	2772	3121	48	77
	ScaffoldSimilarity	4143	4454	77	85
DRD2	No memory	576	759	7	2
	Compound similarity	5243	3401	217	60
	IdenticalBMScaffold	6315	6130	118	35
	IdenticalCarbonSkeleton	6069	5498	61	5
	ScaffoldSimilarity	5433	3794	155	19

The generated compounds were compared with known actives. If the nearest neighbor Tanimoto similarity (using count-based ECFP6) to known actives was larger than 0.4, or if a fragment from a generated compound formed an MMP relationship with a known active it was considered an analog

For HTR1A, the RL with no memory unit generated a total of 1726 ECFP6-based analogs of the training set of the predictive model and 1584 ECFP6 analogs of the validation and test set. In comparison, using the memory-assisted RL at least 2734 analogs to the training set and 2742 analogs to the validation and test set were obtained. Interestingly, the number of MMP analogs did not correlate with the number of generated ECFP6 analogs. In the case of RL with no memory unit, 70 MMP analogs of the training and 69 MMP analogs of the test set were generated. Most MMP analogs were generated using the compound similarity memory unit; 110 MMP analogs of the training set and 97 MMP analogs of the validation and test set. For the identical BM scaffold and the identical carbon skeleton memory unit, the number of MMP analogs of the training set decreased to 57 and 48, respectively. On the other hand, the number of MMP analogs of the test set increased slightly to 89 and 77 for both memory units.

**Fig. 3:**
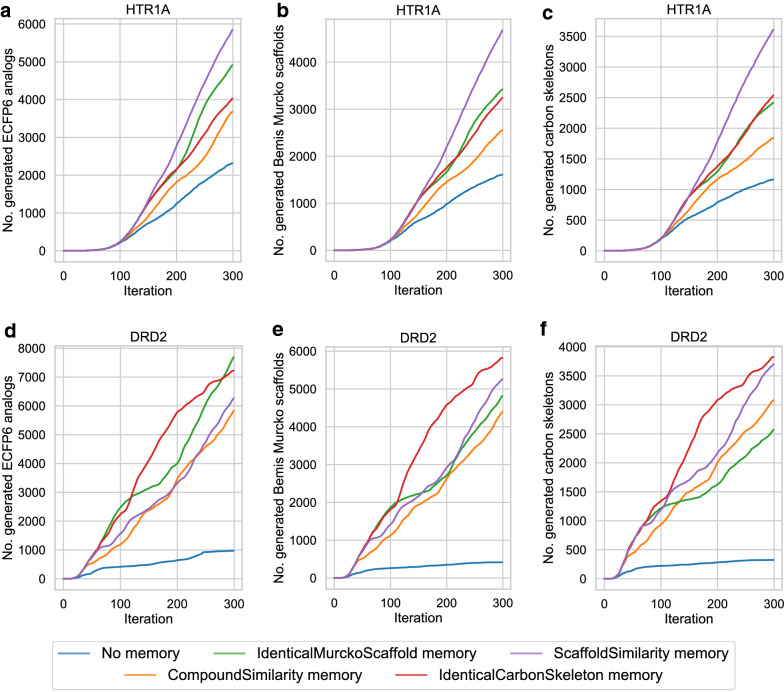
ECFP6 analog generation during reinforcement learning. In **a**-**c** the HTR1A QSAR model was used. In **d**-**f** the DRD2 model was used. **a** and **d** show the number of generated ECFP6 analogs. Compounds with a prediction score of at least 0.7 and Tanimoto similarity (count-based ECFP6) to the nearest neighbor of known actives of at least 0.4 were considered ECFP6 analogs. **b** and **e** show the number of unique BM scaffolds of the generated ECFP6 analogs. **c** and **f** show the number of unique carbon skeletons of the generated ECFP6 analogs

**Fig. 4:**
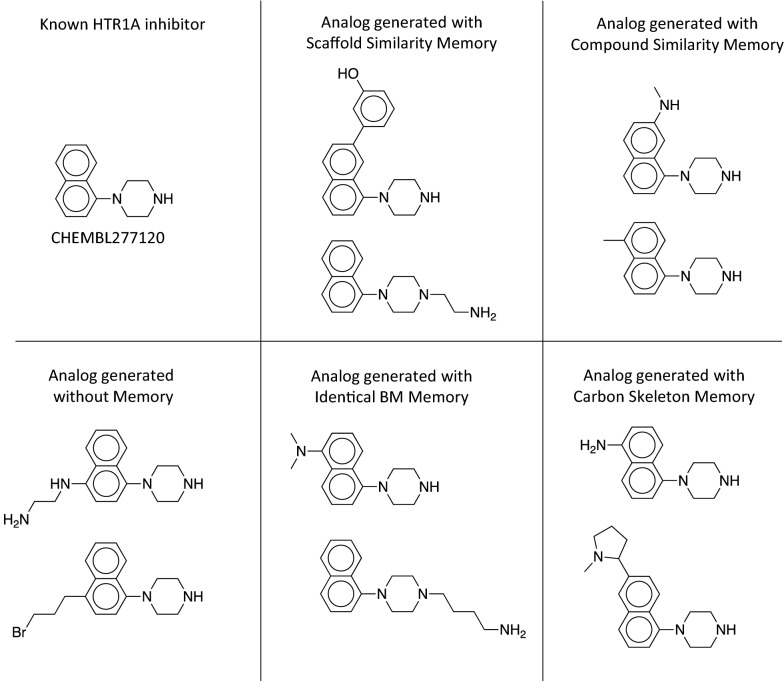
Examples of generated HTR1A-analogs. Shown are three generated examples for the HTR1A optimization using no memory unit and the different memory units, respectively. The closest MMP analog is shown in the upper left

In the case of the DRD2 predictor, the RL without a memory generated 576 ECPF6 analogs to the training set and 759 ECFP6 analogs to the validation and test set. They correspond only to the seven and two MMP analogs, respectively. The identical BM scaffold and the identical carbon skeleton memory showed the largest increase in the number of generated ECFP6 analogs; it increased by more than tenfold in case of the training set and more than sevenfold in the case of the validation and test set, respectively. Importantly, also the number of MMP analogs to the training and test set increased. In the case of the identical BM scaffold memory unit, 118 MMP analogs to the training set and 35 MMP analogs to the test set have been generated. An even higher number of MMP analogs was generated using the compound similarity memory unit; 217 MMP analogs to the training set and 60 MMP analogs to the validation and test set. Despite the high number of generated ECFP6 analogs, the identical carbon skeleton memory unit generated only a few MMP analogs; 61 for the training set and five for the test sets. The memory unit utilizing the scaffold similarity generated 155 MMP analogs of the training set and 19 analogs of the test and validation set. For both targets, the memory unit using the compound similarity led to the largest increase in the generation of MMPs with known active compounds, indicating that application of the compound similarity criterion in RL resulted in highest diversity of newly generated compounds. Compared to the standard RL, memory-assisted RL overall led to broader coverage of chemical space including more highly scored compounds and more diversified BM scaffolds and carbon skeletons. Figure 3 shows the difference in the ECFP6 analog generation utilizing the memory unit during the RL. All calculations generated the first ECFP6 analog around iteration 10. For both targets, the normal RL showed the lowest rate at which ECFP6 analogs were generated. Memory-assisted RL generated ECFP6 analogs at a higher rate. In the case of HTR1A, all memory types generated analogs at a similar rate. The large number of generated ECFP6 analogs also resulted in a larger number of BM scaffolds and carbon skeletons. For DRD2, RL without a memory unit showed a very low rate at which ECFP6 analogs were generated. Between the iteration 100 and 300, only 500 ECFP6 analogs were generated with a predicted activity larger or equal 0.7, despite sampling 20,000 SMILES. This also resulted in a very small number of generated BM scaffold and carbon skeletons for DRD2 (Fig. 3e, f), which illustrates the so-called policy collapse. RL produced highly scoring compounds; however, it did not explore different regions of chemical space. On the other hand, RL with the memory units produced many more ECFP6 analogs with more diverse BM scaffolds and carbon skeletons. By design of the memory unit, different models did not receive a reward when they sampled more than 25 similar compounds. This forced the generative models to explore different regions of chemical space. Similarity measurement of the memory unit determined the directions in which chemical space was further explored. As a consequence, all memory types yielded a significant increase in the rate at which different scaffolds were generated using RL. Exemplary compounds generated using the HTR1A classifier and their MMP analogs are shown in Fig. 4. All RL methods generated analogs for the same experimental validated ligand. RL without a memory unit generated analogs with a linear side chain in para-position to the piperazine with two different characteristics including an aliphatic side chain containing bromine and a more polar side chain having a primary and secondary amine. The analogs generated with the memory unit showed substitutions at different sides. The first exemplary analog of the scaffold similarity memory unit contains a 2-hydroxy benzene attached to the naphthalene and the second analog a short linear side chain ending in a primary amine attached to the piperazine. The memory unit matching identical BM scaffolds generated an analog similar to the analog produced by the scaffold similarity memory, where a linear side chain with a terminal primary amine is attached to the piperazine. In a second analog, a tertiary amine is added at the naphthalene. The compound similarity memory unit generated analogs where a methyl and a secondary amine is attached to the naphthalene. The memory unit matching carbon skeletons produced two analogs with substituents at the naphthalene including one analog with a primary amine and another with a pyrrolidine.

**Fig. 5:**
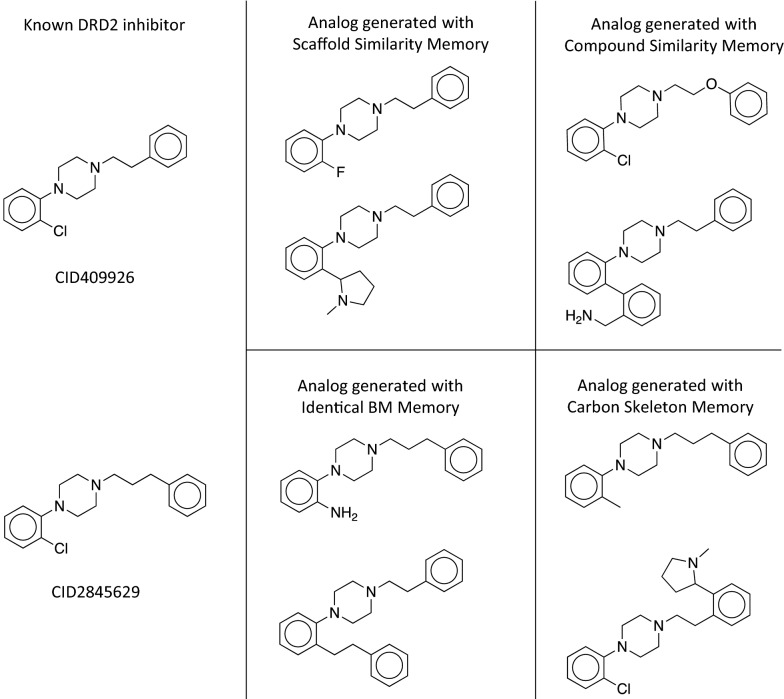
Examples of generated DRD2-analogs. Shown are generated examples for the DRD2 optimization using the different memory units. The known DRD2 inhibitors are shown on the left. Compounds for RL without a memory unit are not shown because it did not generate compounds establishing single cut MMPs with the inhibitors

Eight examples of DRD2 analogs generated with memory units are shown in Fig. 5. Similar to the HTR1A examples, the generated analogs show different types of modifications such as changes in linear chains, functional groups, or ring substituents compared to the known ligand. In the first analog produced by the scaffold similarity memory, identical BM memory, and the carbon skeleton memory unit, the chlorine is replaced with fluorine, a primary amine, or a methyl group. The compound similarity memory unit retains the chlorine but introduces an ether group in the linker between the piperidine and the benzene. The second example for the scaffold similarity memory reveals a change of the chlorine to a 1-methyl pyrrolidine. Also, the compound similarity and the identical BM scaffold memory unit extended the known scaffold by replacing the chlorine with benzene. The carbon skeleton memory unit extended the scaffold on the other side of the compound by adding a 1-methyl pyrrolidine to the left benzene. These examples illustrate how the generative model with memory unit can retrieve known scaffolds of experimentally validated ligands and also extend their scaffold in various ways.

### Influence of parameters

**Fig. 6:**
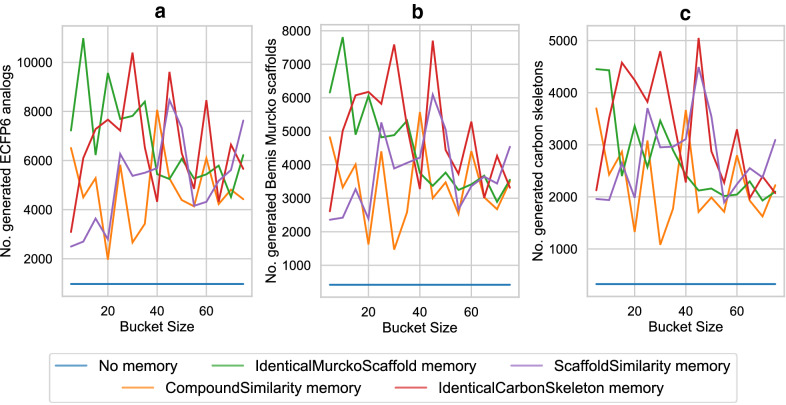
ECFP6 analog generation during reinforcement learning with different bucket sizes. The different bucket sizes do not apply for the RL without a memory unit. In all figures the DRD2 QSAR model was used. **a** shows the number of generated ECFP6 analogs. Compounds with a prediction score of at least 0.7 and Tanimoto similarity (count-based ECFP6) to the nearest neighbor of known actives of at least 0.4 were considered ECFP6 analogs. **b** shows the number of unique BM scaffolds of the generated ECFP6 analogs. **c** shows the number of unique carbon skeletons of the generated ECFP6 analogs

For a deeper understanding of this methodology, we also evaluated the influence of different parameters of the memory unit on the DRD2 analog generation. We benchmarked the effect of the bucket size, the Tanimoto similarity threshold at which compounds are placed in the same bucket, and different output modes. Different bucket sizes ranging from 5 to 75 with an increment of 5 were evaluated. With the exception of matching the identical BM scaffold, every memory unit showed a slight tendency to generate more ECFP6 analogs with increasing bucket size (Fig. 6a). The number of generated BM scaffolds and carbon skeletons show a similar behavior (Fig. 6b, c). For the two memory units measuring the compound and the scaffold similarities, we also assessed the influence of the similarity threshold at which compounds were considered to be similar and assigned to the bucket. For this experiment, we set the bucket size to 25 and evaluated similarity threshold values between 0.3 and 0.9 with an increment of 0.1. The results are shown in Additional file 1: Figure S1. The compound similarity memory units displayed a very clear tendency to generate more ECFP6 analogs for higher similarity values. However, the number of BM scaffolds and carbon skeletons did not significantly increase at similarity thresholds higher than 0.6. The scaffold similarity memory unit generated the maximum number of ECFP6 analogs at a similarity threshold of 0.6. At higher similarity thresholds the number of ECFP6 analogs decreased again. This also applied to the number of BM scaffolds and carbon skeletons.

In addition to these two different parameters we also analyzed two different variants of the memory unit with a different output function. Instead of just giving a binary output of 1 if the bucket is not full and 0 if it is, we tried to smoothen this output for a partially filled bucket using a linear and a sigmoid function. In both cases, the output of the memory unit was reduced inversely proportional to the number of compounds present in a bucket. The linear output mode can be defined as follows:

6 $$M\left( c \right) = 1 - \frac{\# Compounds in Buckets}{Bucket size }$$

The sigmoid is defined as:

7 $$M\left( c \right) = 1 - \frac{1}{{1 + e^{{ - \left( {\frac{{\frac{\# Compounds in buckets}{Bucket size }*2 - 1}}{0.15}} \right)}} }}$$

For both variations of the output mode we repeated the DRD2 experiment with a bucket size of 25 and a Tanimoto similarity value of 0.6. The results are presented in Additional file 1: Figure S2. Both output modes showed a slight decrease the number of ECFP6 analogs, BM scaffolds and carbon skeletons and were therefore not taken into further consideration in combination with different parameters.

### Comparison with other methods for increasing diversity

**Fig. 7:**
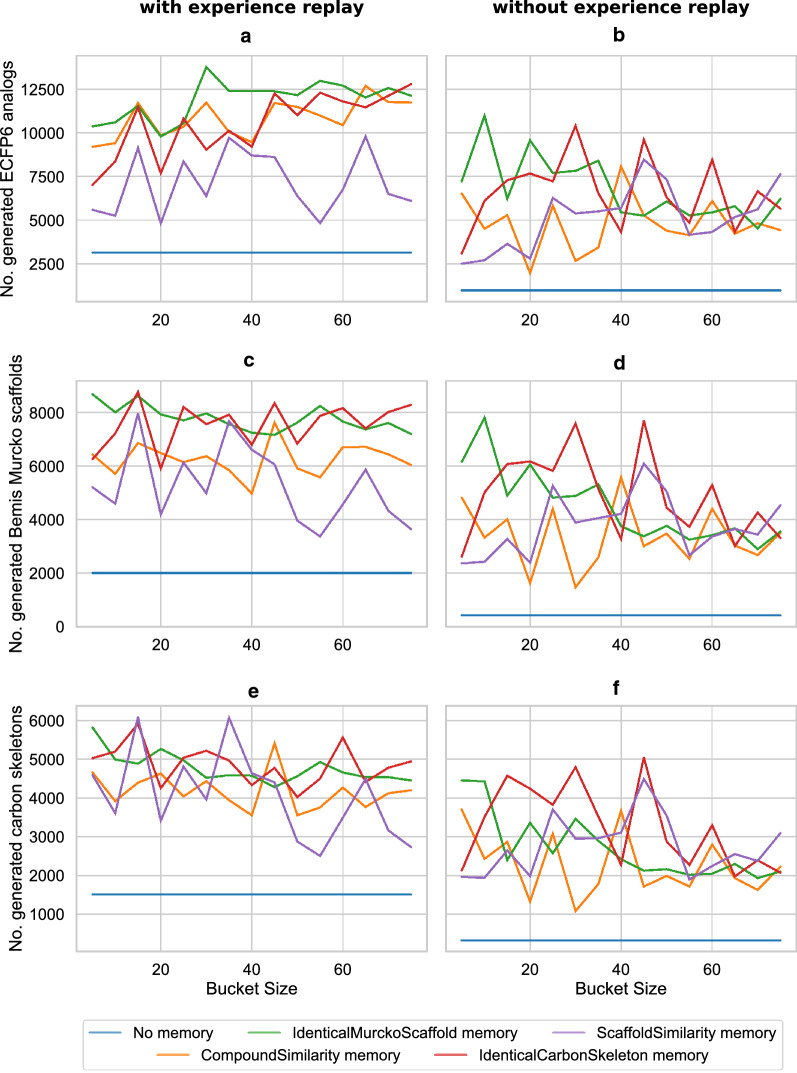
ECFP6 analog generation during reinforcement learning with experience replay and different bucket sizes. In all figures the DRD2 QSAR model was used. (**a**), (**c**) and (**e**) display experiments with experience replay. (**b**), (**d**) and (**f**) experiments without experience replay. **a**, **b** show the number of generated ECFP6 analogs. Compounds with a prediction score of at least 0.7 and Tanimoto similarity (count-based ECFP6) to the nearest neighbor of known actives of at least 0.4 were considered ECFP6 analogs. **c**, d show the number of unique BM scaffolds of the generated ECFP6 analogs. **e, f** show the number of unique carbon skeletons of the generated ECFP6 analogs. In all panels the RL without a memory (blue line) is not affected by the bucket size as this parameter is not present

We also compared the memory unit to another well-established method to increase the diversity, the experience replay [53] method. The basic idea behind experience replay is to learn from highly scoring compounds multiple times. For the experience replay, all generated compounds and their scores were saved. After each iteration of the RL, eight compounds were randomly sampled from the list of saved compounds and the model receives a reward for these compounds. The probability at which the compound were sampled was directly proportional to the saved scores. To evaluate the effect of the experience replay on the methodology, we repeated the previously described experiment with varying bucket sizes. The results are shown in Fig. 7. The blue lines in Fig. 7a, c and e refer to the number of ECFP6 analogs, BM scaffolds and carbon skeletons using RL with experience replay and without the memory unit. They are all below the results of the RL with the different memory units and without experience replay in Fig. 7b, d and f, demonstrating that using the memory unit alone achieves a larger diversity than using experience replay alone. At the same time, when both experience replay and the memory unit are used, the number of ECFP6 analogs, BM scaffolds and carbon skeletons are always larger than the respective result using the memory unit without experience replay. This result suggests that experience replay in combination with the memory unit can achieve an even larger molecular diversity. This is probably because experience replay presents multiple highly scored compounds to the model. The model then uses these compounds as new starting points for searching the chemical space. This way, when the memory unit alters the scoring and forces the model to search for new compounds, the model can start from multiple highly scored compound instead from just the last one.

In addition to the experience replay, we examined different values for the “temperature”, which is used by the RNN during the sampling of the individual tokens. Values larger than 1.0 increase the randomness while generating a SMILES string by smoothing the output distribution of each individual character being sampled. For REINVENT without a memory unit, we examined temperature values ranging from 1.0 to 2.0 with a 0.25 increment and from 2.0 to 10.0 with a 1.0 increment. For a direct comparison with the memory unit, a constant temperature of 1.0 was applied in all cases. The results for different temperature values are shown in Additional file 1: Figure S3. Temperature values greater than 2.0 led prohibited the generation of analogs. Temperatures equal to or greater than 4.0 prevented the generation of valid SMILES. Only the temperature value of 1.25 resulted in a significant increase in the number of generated ECFP6 analogs, BM scaffolds and carbon skeletons. Higher temperature values did not yield any significant increase in the number generated analog. It is important to note that even with the optimized value temperature of 1.25 the number of generated ECFP4 analogs, BM scaffolds and carbon skeletons was only ~ 50% compared to any memory assisted RL. These results are in line with recommendations resulting from other studies [20, 54] not to modify the temperature values because the SMILES syntax is prone to errors with increasing temperature.

The proposed memory unit is a passive memory unit into which compounds only can get added. A different type of memory unit exist in the architectures calles differential neural computers (DNC). A DNC add a memory unit into which the generative model can read and write accorinding to it’s learned parameters. Conceptionally the memory unit is an extension to the memory in common RNN cells like the LSTM.

## Conclusions

We developed the memory unit to address the common issue in RL that the generated compounds often lack chemical diversity due to the so-called policy collapse. The memory unit was designed to be easily integrated into RNNS for RL such as REINVENT. With the introduction of the memory unit, the reward function was modified when the generative model created a sufficient number of similar highly scoring compounds. Therefore, the model must create new chemical entities that are dissimilar to the original solution to maximize the reward again. In the proof-of-concept studies, we optimized the LogP for known bioactive compounds. The results of this optimization indicated that memory-assisted RL led to the generation of more highly-scoring compounds compared to the standard RL. A similar increase in the number of generated compounds as well as the number of BM and carbon scaffolds was observed while optimizing compounds for activity prediction towards HTR1A and DRD2. Additionally, the increase in generated compounds also led to an increase in the generation of analogs. This indicates that the introduction of the memory unit did not reduce the ability of the generative model to produce relevant chemical structures. In summary, our findings indicate that the introduction of the memory unit provides a useful and extendable framework for addressing the so-called policy collapse in generative compound design.

## Supplementary information

**Additional file 1: Figure S1.** ECFP6 analog generation during reinforcement learning with different similarity thresholds. **Figure S2.** ECFP6 analog generation during reinforcement learning with different output modes. **Figure S3.** ECFP6 analog generation during reinforcement learning without memory units and different temperatures

## Data Availability

The data used in this study is publicly available ChEMBL data and ExCAPE data, the algorithm published in this manuscript and the used prepared datasets are made available via GitHub, https://github.com/tblaschke/reinvent-memory.

## Abbreviations

AUC: Area Under Curve
BM: Bemis-Murcko
DRD2: Dopamine Receptor D2
ECFP: Extended-Connectivity Fingerprint
F1: F-measure: harmonic mean of the precision and recall
GAN: Generative Adversarial Networks
GRU: Gated Recurrent Units
HTR1A: 5-Hydroxytryptamine Receptor 1A
MCC: Matthews Correlation Coefficient
MMP: Matched Molecular Pair
NLL: Negative Log-Likelihood
RL: Reinforcement Learning
RNN: Recurrent Neural Networks
ROC: Receiver Operating Characteristic
SMILES: Simple Molecular Input Line Entry System
