# Supplementary material for: Memory-assisted reinforcement learning for diverse molecular de novo design
Source: J Cheminform. 2020 Nov 10;12:68. doi: 10.1186/s13321-020-00473-0 (PMC7654024; doi:10.1186/s13321-020-00473-0)
Supplement: Supplementary file 1 — Additional file 1: Figure S1. ECFP6 analog generation during reinforcement learning with different similarity thresholds. Figure S2. ECFP6 analog generation during reinforcement learning with different output modes. Figure S3. ECFP6 analog generation during reinforcement learning without memory units and different temperatures [file 13321_2020_473_MOESM1_ESM.docx]

# Supplementary Figures: Memory-assisted reinforcement learning for diverse molecular *de novo* design

Thomas Blaschke^§^, Ola Engkvist^§^, Jürgen Bajorath^€^, Hongming Chen^¥,*^

§ Hit Discovery, Discovery Sciences, R&D, AstraZeneca Gothenburg, Sweden

¥ Centre of Chemistry and Chemical Biology, Guangzhou Regenerative Medicine and Health-Guangdong Laboratory, Science Park, Guangzhou, China

€ Department of Life Science Informatics, B-IT, LIMES Program Unit Chemical Biology and Medicinal Chemistry, Rheinische Friedrich-Wilhelms-Universität, Endenicher Allee 19c, D-53115 Bonn, Germany

*Corresponding author: [chen_hongming@grmh-gdl.cn](mailto:chen_hongming@grmh-gdl.cn)


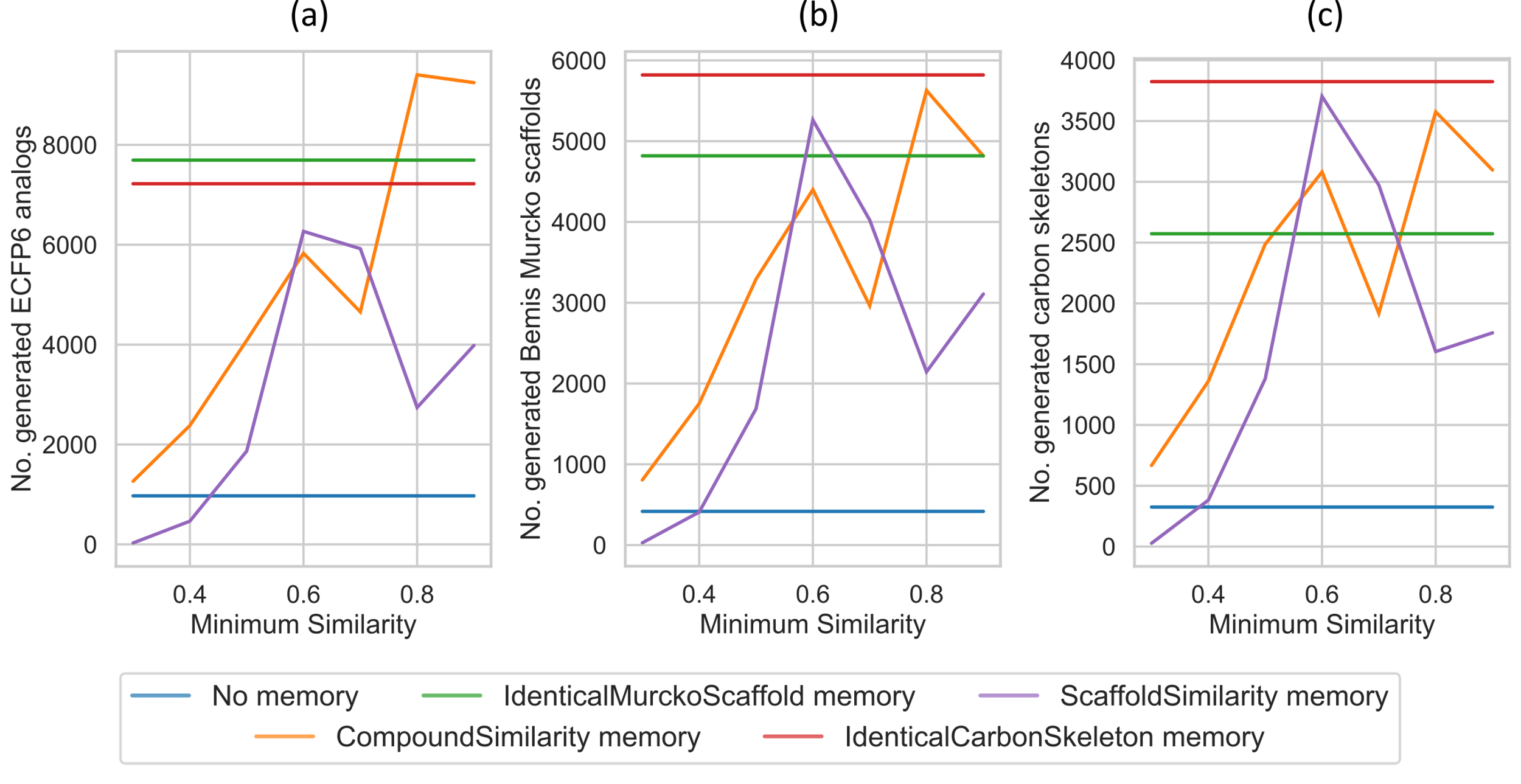


**Figure S1. ECFP6 analog generation during reinforcement learning with different similarity thresholds.** In all figures the DRD2 QSAR model was used. The similarity threshold only applies to the CompoundSimilarity memory and the ScaffoldSimilarity memory. (a) shows the number of generated ECFP6 analogs**.** Compounds with a prediction score of at least 0.7 and Tanimoto similarity (count-based ECFP6) to the nearest neighbor of known actives of at least 0.4 were considered ECFP6 analogs. (b) shows the number of unique BM scaffolds of the generated ECFP6 analogs. (c) shows the number of unique carbon skeletons of the generated ECFP6 analogs.


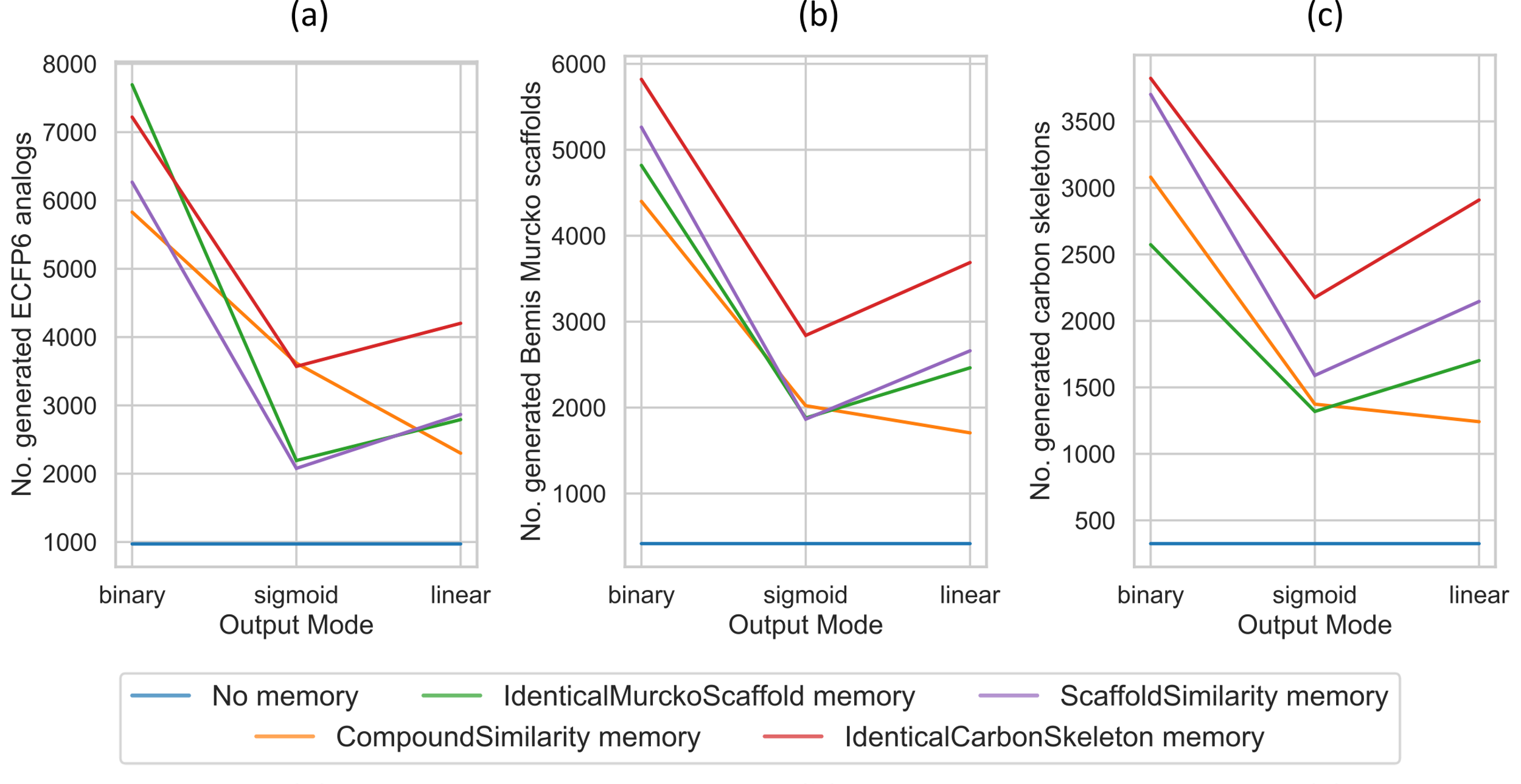


**Figure S2. ECFP6 analog generation during reinforcement learning with different output modes.** In all figures the DRD2 QSAR model was used. (a) shows the number of generated ECFP6 analogs**.** Compounds with a prediction score of at least 0.7 and Tanimoto similarity (count-based ECFP6) to the nearest neighbor of known actives of at least 0.4 were considered ECFP6 analogs. (b) shows the number of unique BM scaffolds of the generated ECFP6 analogs. (c) shows the number of unique carbon skeletons of the generated ECFP6 analogs.


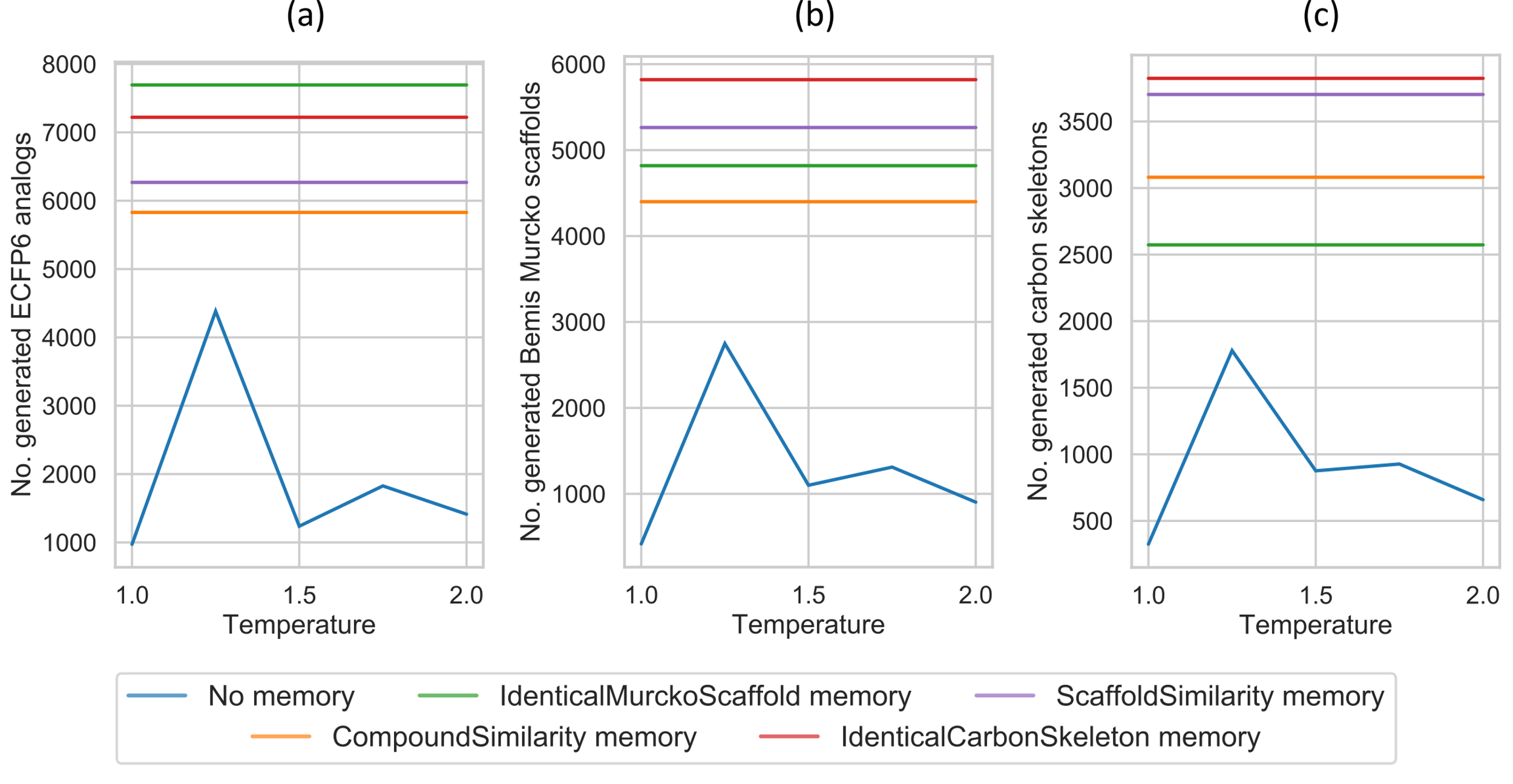


**Figure S3. ECFP6 analog generation during reinforcement learning without memory units and different temperatures.** The temperature value does not apply to RL with any memory. Their values are only provided for reference. In all figures the DRD2 QSAR model was used. (a) shows the number of generated ECFP6 analogs**.** Compounds with a prediction score of at least 0.7 and Tanimoto similarity (count-based ECFP6) to the nearest neighbor of known actives of at least 0.4 were considered ECFP6 analogs. (b) shows the number of unique BM scaffolds of the generated ECFP6 analogs. (c) shows the number of unique carbon skeletons of the generated ECFP6 analogs.
